# Developing and Validating Nomogram to Predict Severe Postpartum Hemorrhage in Women With Placenta Previa Undergoing Cesarean Delivery: A Multicenter Retrospective Case-Control Study

**DOI:** 10.3389/fmed.2021.789529

**Published:** 2022-02-11

**Authors:** Xiaohe Dang, Li Zhang, Yindi Bao, Jie Xu, Hui Du, Shaoshuai Wang, Yanyan Liu, Dongrui Deng, Suhua Chen, Wanjiang Zeng, Ling Feng, Haiyi Liu

**Affiliations:** ^1^Department of Obstetrics and Gynecology, Tongji Hospital, Tongji Medical College, Huazhong University of Science and Technology, Wuhan, China; ^2^Department of Obstetrics and Gynecology, The Central Hospital of Wuhan, Wuhan, China; ^3^Department of Obstetrics, Renmin Hospital of Wuhan University, Wuhan, China; ^4^Department of Obstetrics, Xianning Central Hospital, The First Affiliated Hospital of Hubei University of Science and Technology, Xianning, China; ^5^Department of Obstetrics, Maternal and Child Health Hospital of Hubei Province, Tongji Medical College, Huazhong University of Science and Technology, Wuhan, China

**Keywords:** placenta previa, severe postpartum hemorrhage, multivariate logistic regression, prediction model, nomogram

## Abstract

**Objective:**

Developing and validating nomogram to predict severe postpartum hemorrhage (SPPH) in women with placenta previa (PP) undergoing cesarean delivery.

**Methods:**

We conducted a multicenter retrospective case-control study in five hospitals. In this study, 865 patients from January, 2018 to June, 2020 were enrolled in the development cohort, and 307 patients from July, 2020 to June, 2021 were enrolled in the validation cohort. Independent risk factors for SPPH were obtained by using the multivariate logistic regression, and preoperative nomogram and intraoperative nomogram were developed, respectively. We compared the discrimination, calibration, and net benefit of the two nomograms in the development cohort and validation cohort. Then, we tested whether the intraoperative nomogram could be used before operation.

**Results:**

There were 204 patients (23.58%) in development cohort and 80 patients (26.06%) in validation cohort experienced SPPH. In development cohort, the areas under the receiver operating characteristic (ROC) curve (AUC) of the preoperative nomogram and intraoperative nomogram were 0.831 (95% *CI*, 0.804, 0.855) and 0.880 (95% *CI*, 0.854, 0.905), respectively. In validation cohort, the AUC of the preoperative nomogram and intraoperative nomogram were 0.825 (95% *CI*, 0.772, 0.877) and 0.853 (95% *CI*, 0.808, 0.898), respectively. In the validation cohort, the AUC was 0.839 (95% CI, 0.789, 0.888) when the intraoperative nomogram was used before operation.

**Conclusion:**

We developed the preoperative nomogram and intraoperative nomogram to predict the risk of SPPH in women with PP undergoing cesarean delivery. By comparing the discrimination, calibration, and net benefit of the two nomograms in the development cohort and validation cohort, we think that the intraoperative nomogram performed better. Moreover, application of the intraoperative nomogram before operation can still achieve good prediction effect, which can be improved if the severity of placenta accreta spectrum (PAS) can be accurately distinguished preoperatively. We expect to conduct further prospective external validation studies on the intraoperative nomogram to evaluate its application value.

## Introduction

The placenta previa (PP) is defined as placenta complete or partial covering the internal orifice of cervix (1), the prevalence of PP in obstetrics is about 0.3–1.5% (2). The PP is a major risk factor for severe postpartum hemorrhage (SPPH), which is the leading cause of maternal and neonate mortality and morbidity worldwide (3, 4). In the United States, SPPH accounts for 11% of maternal deaths (3, 5). In China, the incidence of SPPH increased from 0.62% in 2016 to 0.93% in 2018 (6), and the proportion of maternal deaths due to SPPH is increasing, rather than decreasing (7).

The risk prediction tools for SPPH have been established based on previous research of risk factors (8), and researchers developed prediction models for SPPH in women with PP (9–12). However, they cannot be recommended for clinical at present due to lack of studies on the performance, impact, and effectiveness of these models (13). Future research is needed to develop models with strong applicability (14). Therefore, we aim to develop and validate a new nomogram to predict SPPH in women with PP undergoing cesarean delivery by a multicenter retrospective case-control study.

## Materials and Methods

### Research Centers and Patients

**Figure 1:**
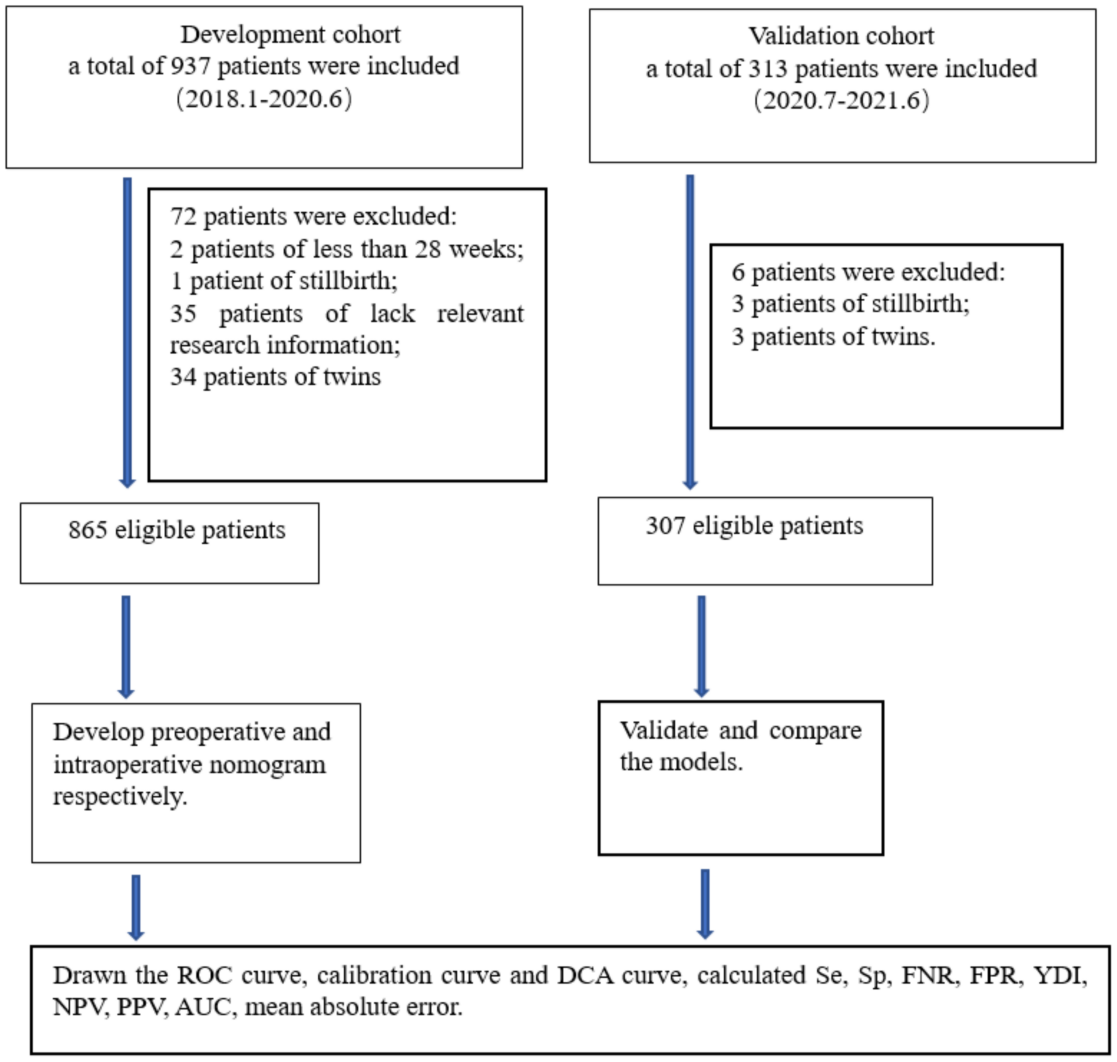
Flow chart for study design. ROC, receiver operating characteristic; DCA, decision curve analysis; Se, sensitivity; Sp, specificity; FNR, false negative rate; FPR, false positive rate; PPV, positive predictive value; NPV, negative predictive value; AUC, area under the receiver operating characteristic curve; YDI, Youden's index.

We aimed to conduct a multicenter retrospective case-control study in five centers. The research centers included are Tongji Hospital, Renmin Hospital of Wuhan University, Maternal and Child Health Hospital of Hubei Province, Xianning Central Hospital, and the Central Hospital of Wuhan. They are all tertiary hospital in Hubei Province, China. Patients were retrieved through electronic medical record systems in each hospital. The inclusion criteria were patients with PP who underwent cesarean delivery after 28 weeks of gestation between January, 2018 and June, 2021. The exclusion criteria were induced labor, twins, vaginal delivery, preoperative stillbirth, and incomplete ultrasound and clinical information. A total of 1,250 patients were retrieved, 78 patients were excluded according to the inclusion criteria and exclusion criteria, the remaining 1,172 patients were enrolled in the study. Then, 865 patients from January, 2018 to June, 2020 were divided into the development cohort, and 307 patients from July, 2020 to June, 2021 were divided into the validation cohort. The research centers, principal investigators, and the number of patients recruited in the centers are listed in Appendix Table 1. The process of inclusion, exclusion, and grouping of the patients is shown in Figure 1.

### Data Collection

We collected the general characteristics and clinical manifestations of patients, such as maternal age, *in-vitro* fertilization (IVF), intracytoplasmic sperm injection (ICSI), delivery gestational age (DGA), fibroids, complications (such as hypertensive disorders of pregnancy, diabetes mellitus, intrahepatic cholestasis of pregnancy, pulmonary hypertension, and cardiac insufficiency), gravida, parity, number of prior cesarean delivery (CDs) and abortion, history of intrauterine operations, postpartum hemorrhage (PPH), myomectomy and abdominal operations, preoperative bleeding (PB), hemoglobin (HGB), and use of tocolytics. The ultrasound information, such as fetal position, anterior placenta (AP), PP, placenta accreta spectrum (PAS), placenta sinuses (PS), blood flow signal (BFS) in lower uterine segment were collected. Moreover, we collected the intraoperative conditions and maternal and neonate outcomes, such as vascular engorgement (VE) in lower uterine segment, the anterior wall of the uterus adheres to the surrounding tissues, muscular layer thinning, PAS, arterial balloon presetting, tourniquet or serrata forceps, uterine artery ligation, suture hemostasis, tamponade balloon or gauze in uterine, hysterectomy, intraoperative blood loss, red blood cell (RBC) transfusion unit, cell salvage, use other blood products (platelets, fibrin, plasma, etc.), postoperative bleeding, transfer to ICU, postoperative hospital stay, neonate birth weight, 1-min Apgar score, 5-min Apgar score, and transfer to neonatology.

### Variable Definitions

Referring to the recommendations of the Society of Obstetricians and Gynecologists of Canada (1), placenta covering the internal orifice of cervix was defined as PP, and placenta lower than 2 cm away from the internal orifice of cervix was defined as low-lying placenta (LL). According to the Federation International of Gynecology and Obstetrics (FIGO) consensus guidelines (15), the PAS disorders were divided into three categories: crete, increta, and percreta. Due to the low rate of placenta crete and percreta reported by preoperative ultrasound, we did not distinguish the severity of PAS preoperatively and only classified with and without PAS, and we classified the severity of PAS according to intraoperative conditions. The comparison of preoperative ultrasound and intraoperative assessment of the severity of PAS is shown in Appendix Table 2. Following the American protocol for obstetric mass transfusion (16) and definition of SPPH in other study (17), we defined SPPH as total blood loss ≥ 1,500 ml or RBC transfusion ≥4U between cesarean delivery and 24 h before and after cesarean delivery (the intraoperative cell savage volume was converted into RBC transfusion units, 200 ml = 1U). Combining blood loss volume and RBC transfusion unit as the definition of SPPH is helpful to avoid misjudgment of outcome caused by the underestimation of blood loss and insufficient blood transfusion due to inadequate blood supply.

In our study, preoperative and postoperative bleeding was defined as excessive bleeding that required urgent management. The intrauterine operations referred to diagnostic curettage, endometrium polyp extirpation, uterus mediastinum surgery, and uterine cavity adhesion decomposition. Our definition of abdominal operations excluded myomectomy and cesarean section. If the placenta mostly covered the anterior wall of uterus, it was defined as the anterior placenta.

### Statistical Analyses

The quantitative variables in this study were not normally distributed and were represented as P50 (P10, P90), and the categorical variables were described as *n* (%). We used the chi-square test or Fisher's exact test to preliminarily screened risk factors of SPPH in development cohort, and adopted multivariate stepwise forward logistic regression to screen independent risk factors. Based on the independent risk factors, we established the preoperative nomogram and intraoperative nomogram, respectively. Nomogram is a visual graphical tool for quantitative calculation, which makes the complicated regression results easy to calculate (18). Then, we have drawn receiver operating characteristic (ROC) curve for the two nomograms in development cohort, the cut-off point was determined when the Youden's index (YDI) was maximum (YDI = sensitivity (Se) +specificity (Sp) – 1).

In validation cohort, we calculated the total points of each patient using the nomograms. The patient was predicted to experience SPPH if the total points exceeded the cut-off value. The actual occurrence of SPPH as the gold standard, and the predicted result as the test value to calculate the Se, Sp, YDI, false negative rate (FNR), false positive rate (FPR), positive predictive value (PPV), negative predictive value (NPV), and draw the ROC curve, decision curve analysis (DCA) curve, and calibration curve of each nomogram. We used the DeLong's test to compare the areas under the ROC curve (AUC) between models. Calibration curves were drawn by bootstrap validation with 1,000 repetitions. In this study, the incidence of SPPH in development cohort was 23.58%, so the population prevalence was 0.236 when DCA curves were drawn.

Statistical analyses were performed using the IBM SPSS statistics (version 22.0, IBM, NY, USA), R software (version 4.1.0, Austria), and MedCalc (version 12.7.0, Belgium). All tests were two-sided, and *p* < 0.05 was considered statistically significant.

## Results

In our study, there were 204 patients (23.58%) in the development cohort and 80 patients (26.06%) in the validation cohort experienced SPPH. Between the development and validation cohort, there were statistical differences in the proportion of PP (*p* = 0.022), preoperative HGB < 90 g/L (*p* = 0.012), PB (*p* = 0.048), the anterior wall of the uterus adheres to the surrounding tissue (*p* = 0.044) and the severity of PAS found during operation (*p* = 0.009), and there were no statistical differences in other factors. Patients characteristics are summarized in Table 1. In both cohorts, there were statistical differences in the rate of artery balloon presetting, tourniquet or serrata forceps, suture hemostasis, uterine artery ligation, tamponade balloon or gauze in uterine, hysterectomy and postoperative hospital stay, the rate of premature birth, low birth weight, neonatal asphyxia, and transfer to neonatology between the SPPH and non-SPPH group, the values of *p* were all <0.05. In the development cohort, there were statistical differences in the rate of transfer to ICU (*p* < 0.001) and postoperative bleeding (*p* < 0.001) between SPPH and non-SPPH group. However, there were no statistical differences in the rate of transfer to ICU (*p* = 0.067) and postoperative bleeding (*p* = 0.167) between the SPPH and non-SPPH group in the validation cohort, we considered that might be related to the small sample size in validation cohort. The intraoperative hemostatic measures are shown in Appendix Table 3, and the maternal and neonate outcomes are shown in the Appendix Table 4.

**Table 1:** Baseline characteristics of patients in the development cohort and validation cohort.

**Characteristics**	**Total (*n* = 1,172)**	**Development cohort (*n* = 865)**	**Validation cohort (*n* = 307)**	***P-*value**
**Preoperative characteristics**				
Maternal age ≥35 years-n (%)	375 (32.00)	277 (32.02)	98 (31.92)	0.974
IVF or ICSI-n (%)	139 (11.86)	107 (12.37)	32 (10.42)	0.365
Delivery gestational age (DGA) <37w-n (%)	658 (56.14)	492 (56.88)	166 (54.07)	0.394
Fibroids-n (%)	86 (7.34)	64 (7.40)	22 (7.17)	0.893
Complications (HDP, DM, ICP, PH, CI)-n (%)	286 (24.40)	210 (24.28)	76 (24.76)	0.867
Gravida-n (%)				0.199
1–2	442 (37.71)	339 (39.19)	103 (33.55)	
3–4	484 (41.30)	351 (40.58)	133 (43.32)	
≥5	246 (20.99)	175 (20.23)	71 (23.13)	
Parity-n (%)				0.361
0	391 (33.36)	290 (33.53)	101 (32.90)	
1	636 (54.27)	475 (54.91)	161 (52.44)	
≥2	145 (12.37)	100 (11.56)	45 (14.66)	
Number of prior CDs-n (%)				0.239
0	655 (55.89)	483 (55.84)	172 (56.03)	
1	437 (37.29)	329 (38.03)	108 (35.18)	
≥2	80 (6.83)	53 (6.13)	27 (8.79)	
Number of prior abortion-n (%)				0.096
0	372 (31.74)	289 (33.41)	83 (27.04)	
1–2	574 (48.98)	417 (48.21)	157 (51.14)	
≥3	226 (19.28)	159 (18.38)	67 (21.82)	
History of vaginal birth-n (%)	295 (25.17)	222 (25.66)	73 (23.78)	0.513
History of intrauterine operations-n (%)	90 (7.68)	68 (7.86)	22 (7.17)	0.694
History of PPH-n (%)	17 (1.45)	15 (1.73)	2 (0.65)	0.278
History of myomectomy-n (%)	21 (1.79)	15 (1.73)	6 (1.95)	0.803
History of abdominal operations-n (%)	148 (12.63)	117 (13.53)	31 (10.10)	0.120
Fetal non-cephalic presentation-n (%)	272 (23.21)	199 (23.01)	73 (23.78)	0.783
Anterior placenta (AP)-n (%)	542 (46.25)	402 (46.47)	140 (45.60)	0.792
Placenta previa type (PPT)-n (%)				0.022
Placenta previa (PP)	809 (69.03)	613 (70.87)	196 (63.84)	
Low-lying placenta (LL)	363 (30.97)	252 (29.13)	111 (36.16)	
Placenta accreta spectrum (PAS) (%)	280 (23.89)	205 (23.70)	75 (24.43)	0.796
Placental sinuses (PS)-n (%)	135 (11.52)	99 (11.45)	36 (11.73)	0.894
Blood flow signal of lower uterine segment (BFS)-n (%)	268 (22.87)	200 (23.12)	68 (22.15)	0.728
Preoperative bleeding (PB)-n (%)	177 (15.10)	120 (13.87)	57 (18.57)	0.048
Preoperative HGB <90 g/l-n (%)	68 (5.80)	59 (6.82)	9 (2.93)	0.012
Tocolytics-n (%)	403 (34.39)	302 (34.91)	101 (32.90)	0.523
**Intraoperative characteristics**				
Lower uterine segment vascular engorgement (VE)-n (%)	410 (34.98)	289 (33.41)	121 (39.41)	0.058
The anterior wall of the uterus adheres to the surrounding tissues-n (%)	128 (10.92)	85 (9.83)	43 (14.01)	0.044
Muscular layer thinning-n (%)	175 (14.93)	125 (14.45)	50 (16.29)	0.438
Placenta accreta spectrum (PAS)-n (%)				0.009
Normal	558 (47.61)	426 (49.25)	132 (43.00)	
Crete	219 (18.69)	142 (16.42)	77 (25.08)	
Increta	366 (31.23)	276 (31.90)	90 (29.32)	
Percreta	29 (2.47)	21 (2.43)	8 (2.60)	

*IVF, in-vitro fertilization; ICSI, intracytoplasmic sperm injection; CD, cesarean delivery; HDP, hypertensive disorders of pregnancy; DM, diabetes mellitus; ICP, intrahepatic cholestasis of pregnancy; PH, pulmonary hypertension; CI, cardiac insufficiency*.

**Figure 2:**
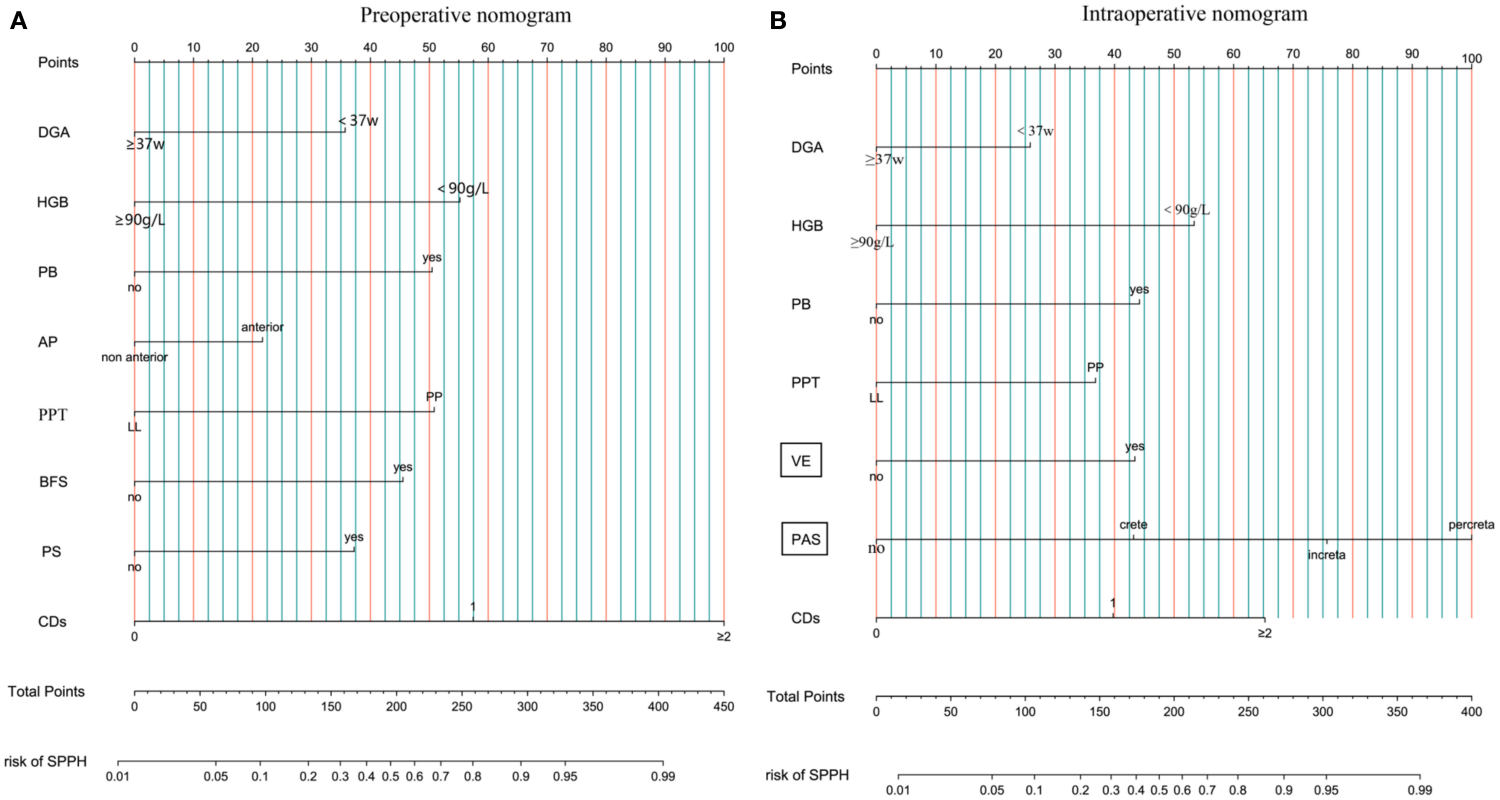
Nomogram. **(A)** Preoperative nomogram. **(B)** Intraoperative nomogram. DGA, delivery gestational age; HGB, hemoglobin; PB, preoperative bleeding; AP, anterior placenta; PPT, placenta previa type; LL, low-lying; PP, placenta previa; BFS, blood flow signal of lower uterine segment; PS, placental sinuses; CDs, number of prior cesarean delivery; VE, vascular engorgement of lower uterine segment; PAS, placenta accrete spectrum; SPPH, severe postpartum hemorrhage.

In the development cohort, among the features which can be detected preoperatively, there were statistical differences in IVF or ICSI (*p* < 0.001), DGA < 37 w (*p* < 0.001), fibroids (*p* = 0.013), gravida (*p* < 0.001), parity (*p* < 0.001), number of prior CDs (*p* < 0.001), number of prior abortion (*p* = 0.004), history of PPH (*p* = 0.015), AP (*p* < 0.001), PP (*p* < 0.001), PAS (*p* < 0.001), PS (*p* < 0.001), BFS (*p* < 0.001), PB (*p* < 0.001), preoperative HGB < 90 g/L (*p* < 0.001), use of tocolytics (*p* < 0.001) between the SPPH and non-SPPH group. Among the features which can be detected intraoperatively, there were statistical differences in VE (*p* < 0.001), the anterior wall of uterus adheres to the surrounding tissues (*p* = 0.001), muscular layer thinning (*p* < 0.001) and the severity of PAS (*p* < 0.001) between the SPPH and non-SPPH group. The results of univariate analysis are shown in Table 2. We used the independent risk factors in the preoperative characteristics screened by using the multivariate logistic regression to establish preoperative nomogram. According to the point of each predictor shown in the preoperative nomogram, the formula is: total points = DGA < 37 w (36 points) + HGB < 90 g/L (55 points) + PB (50.5 points) + AP (22 points) + PP (51 points) + PS (37.5 points) + BFS (45.5 points) + number of CDs (0: 0 point; 1: 57.5 points; ≥2: 100 points). We used the independent risk factors in the preoperative and intraoperative characteristics screened by using the multivariate logistic regression to establish intraoperative nomogram. Due to the intraoperative characteristics included the severity of PAS and VE, the PAS and BFS detected by ultrasound were not included in the regression analysis to avoid inclusion of similar indicators. The formula of the intraoperative nomogram is: total points = DGA < 37 w (25.5 points) + HGB < 90 g/L (53 points) + PB (44.5 points) + VE (43.5 points) + PP (37 points) + PAS (normal: 0 point; crete: 43 points; increta:76 points; percreta:100 points) + number of prior CDs (0: 0 point; 1: 40 points; ≥2: 65 points). The cut-off points of the preoperative nomogram and intraoperative nomogram are 145.25 and 145.75, respectively. The multivariate logistic regression results are reported in Table 3, and the nomograms are shown in Figure 2.

**Table 2:** Univariate analysis in the development cohort.

**Characteristics**	**Total (*n* = 865)**	**Non SPPH group (*n* = 661)**	**SPPH group (*n* = 204)**	***P*-value**
**Preoperative characteristics**				
Maternal age ≥35 years-n (%)	277 (32.02)	214 (32.38)	63 (30.88)	0.690
IVF or ICSI-n (%)	107 (12.37)	97 (14.67)	10 (4.90)	<0.001
Delivery gestational age (DGA) <37w-n (%)	492 (56.88)	331 (50.08)	161 (78.92)	<0.001
Fibroids-n (%)	64 (7.40)	57 (8.62)	7 (3.43)	0.013
Complications (HDP, DM, ICP, PH, CI) -n (%)	210 (24.28)	162 (24.51)	48 (23.53)	0.776
Gravida-n (%)				<0.001
1–2	339 (39.19)	287 (43.42)	52 (25.49)	
3–4	351 (40.58)	262 (39.64)	89 (43.64)	
≥5	175 (20.23)	112 (16.94)	63 (30.88)	
Parity-n (%)				<0.001
0	290 (33.53)	264 (39.94)	26 (12.74)	
1	475 (54.91)	342 (51.74)	133 (65.20)	
≥2	100 (11.56)	55 (8.32)	45 (22.06)	
Number of prior CDs-n (%)				<0.001
0	483 (55.84)	426 (64.45)	57 (27.94)	
1	329 (38.03)	211 (31.92)	118 (57.84)	
≥ 2	53 (6.13)	24 (3.63)	29 (14.22)	
Number of prior abortion-n (%)				0.004
0	289 (33.41)	235 (35.55)	54 (26.47)	
1–2	417 (48.21)	319 (48.26)	98 (48.04)	
≥3	159 (18.38)	107 (16.19)	52 (25.49)	
History of vaginal birth-n (%)	222 (25.66)	178 (26.93)	44 (21.57)	0.125
History of intrauterine operations-n (%)	68 (7.86)	52 (7.87)	16 (7.84)	0.991
History of PPH-n (%)	15 (1.73)	7 (1.06)	8 (3.92)	0.015
History of myomectomy-n (%)	15 (1.73)	12 (1.82)	3 (1.47)	0.982
History of abdominal operations-n (%)	117 (13.53)	89 (13.46)	28 (13.73)	0.924
Fetal non-cephalic presentation-n (%)	199 (23.01)	145 (21.94)	54 (26.47)	0.179
Anterior placenta (AP)-n (%)	402 (46.47)	265 (40.09)	137 (67.16)	<0.001
Placenta previa type (PPT)-n (%)				<0.001
Placenta previa (PP)	613 (70.87)	431 (65.20)	182 (89.22)	
Low-lying placenta (LL)	252 (29.13)	230 (34.80)	22 (10.78)	
Placenta accreta spectrum (PAS)-n (%)	205 (23.70)	134 (20.27)	71 (34.80)	<0.001
Placental sinuses (PS)-n (%)	99 (11.45)	56 (8.47)	43 (21.08)	<0.001
Blood flow signal of lower uterine segment (BFS)-n (%)	200 (23.12)	83 (12.56)	117 (57.35)	<0.001
Preoperative bleeding (PB)-n (%)	120 (13.87)	73 (11.04)	47 (23.04)	<0.001
Preoperative HGB <90 g/L-n (%)	59 (6.82)	29 (4.39)	30 (14.71)	<0.001
tocolytics-n (%)	302 (34.91)	210 (31.77)	92 (45.10)	<0.001
**Intraoperative characteristics**				
Vascular engorgement of lower uterine segment (VE)-n (%)	289 (33.41)	162 (24.51)	127 (62.25)	<0.001
The anterior wall of the uterus adheres to the surrounding tissues-n (%)	85 (9.83)	53 (8.02)	32 (15.69)	0.001
Muscular layer thinning-n (%)	125 (14.45)	58 (8.77)	67 (32.84)	<0.001
Placenta accreta spectrum (PAS)-n (%)				<0.001
Normal	426 (49.25)	398 (60.21)	28 (13.73)	
Crete	142 (16.42)	113 (17.10)	29 (14.22)	
Increta	276 (31.91)	144 (21.79)	132 (64.71)	
Percreta	21 (2.43)	6 (0.91)	15 (7.35)	

*IVF, in-vitro fertilization; ICSI, intracytoplasmic sperm injection; CD, cesarean delivery; HDP, hypertensive disorders of pregnancy; DM, diabetes mellitus; ICP, intrahepatic cholestasis of pregnancy; PH, pulmonary hypertension; CI, cardiac insufficiency; HGB, hemoglobin*.

**Table 3:** Multivariate logistic regression in the development cohort.

**Variables**	***P***	**OR (95%CI)**
**Preoperative prediction model**		
Delivery gestational age (DGA) <37 w	<0.001	2.200 (1.433, 3.380)
Preoperative HGB <90 g/L	<0.001	3.383 (1.785, 6.412)
Preoperative bleeding (PB)	<0.001	3.048 (1.853, 5.013)
Anterior placenta (AP)	0.016	1.614 (1.093, 2.385)
Placenta previa (PP)	<0.001	3.073 (1.794, 5.262)
Placental sinuses (PS)	0.002	2.275 (1.361, 3.805)
Blood flow signal of lower uterine segment (BFS)	<0.001	2.733 (1.796, 4.159)
Number of prior CDs		
0	Reference	Reference
1	<0.001	3.555 (2.342, 5.396)
≥2	<0.001	9.097 (4.532, 18.263)
**Intraoperative prediction model**		
Delivery gestational age (DGA) <37 w	0.004	1.969 (1.244, 3.118)
Preoperative HGB <90 g/l	<0.001	4.053 (2.006, 8.191)
Preoperative bleeding (PB)	<0.001	3.185 (1.841, 5.510)
Placenta previa (PP)	0.001	2.625 (1.478, 4.663)
Vascular engorgement of lower uterine segment (VE)	<0.001	3.122 (2.069, 4.710)
Placenta accreta spectrum (PAS)		
Normal	Reference	Reference
Crete	<0.001	3.105 (1.674, 5.759)
Increta	<0.001	7.274 (4.366, 12.118)
Percreta	<0.001	13.764 (4.244, 44.642)
Number of prior CDs		
0	Reference	Reference
1	<0.001	2.836 (1.815, 4.430)
≥2	<0.001	5.537 (2.599, 11.795)

*HGB, hemoglobin; CD, cesarean delivery*.

**Figure 3:**
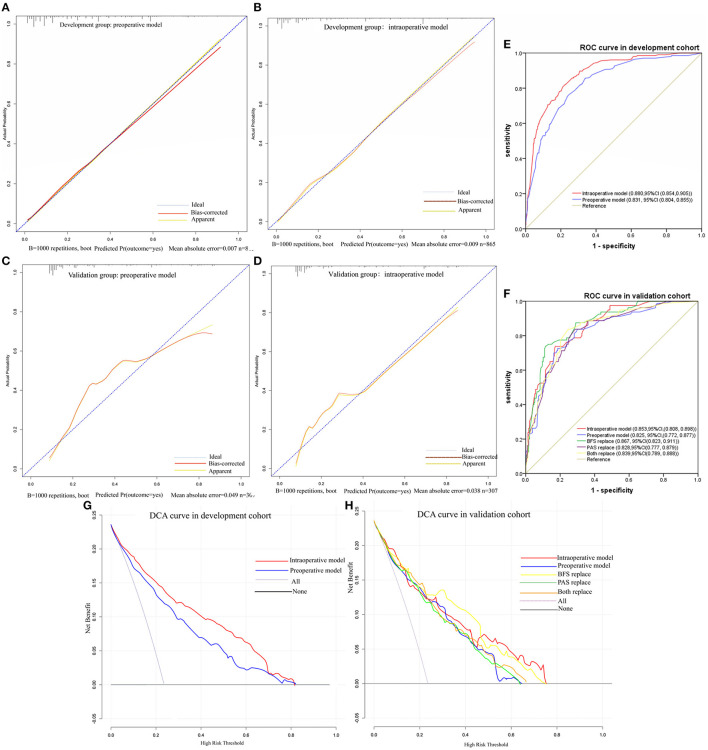
Calibration cures, ROC cures and DCA cures. **(A)** Calibration cure of preoperative model in development cohort. **(B)** Calibration cure of intraoperative model in development cohort. **(C)** Calibration cure of preoperative model in validation cohort. **(D)** Calibration cure of intraoperative model in validation cohort. **(E)** ROC curve in development cohort. **(F)** ROC curve in validation cohort. **(G)** DCA curve in development cohort. **(H)** DCA curve in validation cohort.

The nomograms were first evaluated in the development cohort. The AUC of preoperative nomogram and intraoperative nomogram was 0.831 (95% *CI*, 0.804–0.855) and 0.880 (95% *CI*, 0.854–0.905), respectively and DeLong's test confirmed that there was significant different between the intraoperative and preoperative nomogram (*p* < 0.001). The Se, Sp, YDI, PPV, and NPV of intraoperative nomogram were all higher than the preoperative nomogram, the FNR and FPR of intraoperative nomogram were all lower than the preoperative nomogram. In DCA curves, the intraoperative nomogram obtained more net benefit than the preoperative nomogram in most areas of the risk threshold. The calibration curves of the preoperative nomogram and intraoperative nomogram were all close to the ideal curves. In the validation cohort, the AUC of preoperative nomogram and intraoperative nomogram was 0.825 (95% *CI*, 0.772–0.877) and 0.853 (95% *CI*, 0.808–0.898), respectively. The AUC of the intraoperative nomogram was still higher than the preoperative nomogram, but there was no statistical difference (*p* = 0.117). The Se, NPV, and FPR of intraoperative nomogram were all higher than the preoperative nomogram, but the Sp, YDI, FNR, and PPV of intraoperative nomogram were all slightly lower than the preoperative nomogram. In DCA curves, the intraoperative nomogram obtained more net benefit than the preoperative nomogram in most areas of the risk threshold. In addition, the calibration curve of intraoperative nomogram was closer to the ideal curve than that of preoperative nomogram. By comparing the discrimination, calibration, and net benefit of the intraoperative nomogram and preoperative nomogram in the development and validation cohort, we think that the intraoperative nomogram is more effective than the preoperative nomogram. The test results of the two nomograms are shown in Table 4. The ROC curves, DCA curves, and calibration curves are given in Figure 3.

**Table 4:** Prediction results.

	**Development cohort**		**Validation cohort**				
	**Preoperative nomogram**	**Intraoperative nomogram**	**Preoperative nomogram**	**Intraoperative nomogram**	**Predictors in the intraoperative nomogram were replaced by preoperative ultrasound**		
					**BFS replace** **VE**	**PAS detected in ultrasound replace** **PAS found intraoperatively**	**Both replace**
Cut-off points	145.25	145.75	145.25	145.75	145.75	145.75	145.75
Se (%)	75.98	80.88	72.50	73.75	76.25	65.00	62.50
Sp (%)	75.79	78.82	80.62	78.41	83.70	82.38	85.46
FNR (%)	24.02	19.12	27.50	26.25	23.75	35.00	37.50
FPR (%)	24.21	21.18	19.38	21.59	16.30	17.62	14.54
YDI	0.52	0.60	0.53	0.52	0.60	0.47	0.48
PPV (%)	49.21	54.10	56.86	54.63	62.24	56.52	60.24
NPV (%)	91.09	93.04	89.27	89.45	90.91	86.98	86.61
AUC (95%CI)	0.831 (0.804, 0.855)	0.880 (0.854, 0.905)	0.825 (0.772, 0.877)	0.853 (0.808, 0.898)	0.867 (0.823, 0.911)	0.828 (0.777, 0.879)	0.839 (0.789, 0.888)
P	<0.001		0.117		-	-	-
P^*^	–		–		0.181	0.170	0.478
P^#^	–		–		0.004	0.839	0.283

*Se, sensitivity; Sp, specificity; FNR, false negative rate; FPR, false positive rate; YDI, Youden's index; PPV, positive predictive value; NPV, negative predictive value; AUC, area under the receiver operating characteristic curve; CI, confidence interval; BFS, blood flow signal of lower uterine segment; VE, vascular engorgement; PAS, placenta accreta spectrum*.

* *Compared with when the predictors in the intraoperative nomogram were not replaced*.

# *Compared with the preoperative nomogram*.

To examine whether the intraoperative nomogram can be applied before operation and the effect when used before operation, we performed a predictor replacement test in the validation cohort. Due to VE and the severity of PAS found in operation cannot be obtained before operation, we used BFS and the severity of PAS detected by ultrasound to replace VE and PAS found in operation. For example, if preoperative ultrasound of a patient suggests BFS in the lower uterine segment, VE = 43.5 points should be observed, and the AUC [0.867, 95% *CI*, (0.823, 0.911)], Se (76.25%), Sp (83.70%), YDI (0.60), PPV (62.24%), NPV (90.91%), and DCA were slightly higher than VE was not replaced. And if preoperative ultrasound of a patient suggests placenta increta, PAS = 76 points should be observed, and the AUC [0.828 (95% *CI*, 0.777, 0.879)], Se (65%), YDI (0.47), NPV (86.98%), and DCA were slightly lower than the severity of PAS found intraoperatively was not replaced. The intraoperative nomogram can be used preoperatively only when the two predictors are replaced together, and the AUC [0.839 (95% *CI*, 0.789, 0.888)], Se (62.5%), YDI (0.48), NPV (86.61%), and DCA were slightly lower than the two predictors that were not replaced. But DeLong's test confirmed that there were no statistical differences between the two predictors that were replaced compared with the original intraoperative nomogram (*p* = 0.478) and preoperative nomogram (*p* = 0.283). The test results of predictors that were replaced are shown in Table 4, and the ROC curves and DCA curves are given in Figure 3.

## Discussion

We established nomogram to predict the risk of SPPH in patients with PP undergoing cesarean delivery in this study. We hope that the nomogram can help clinicians identify patients at high risk of SPPH and make adequate preoperative preparations to reduce the intraoperative blood loss and the incidence of serious maternal and neonate complications caused by SPPH. In the following paragraphs, we will discuss the predictors, predictive performance, and application of the nomogram.

We established the preoperative nomogram and intraoperative nomogram according to whether intraoperative factors were included in the multivariate logistic regression analysis. The preoperative nomogram contains eight predictors and the intraoperative nomogram contains seven predictors, of which five predictors are same. The five same predictors are DGA < 37 w, preoperative HGB < 90 g/L, PB, PP, and number of prior CDs, respectively. The difference is that the intraoperative nomogram contains the severity of PAS and VE detected intraoperatively, while the preoperative nomogram contains AP, BFS, and PS detected by ultrasound. It may be related to the poor diagnostic accuracy of PAS by ultrasound and we guess that the AP plus PS may indicate the presence of PAS. Considering the subjective differences of operators in judging the degree of BFS, the size and number of PS and the degree of VE in the lower uterine segment, we divided the three factors into dichotomous variables (yes and no). Moreover, the dichotomous is more convenient for clinical application than the multi-classification made by Kang et al. (11), and has no adverse impact on the prediction effect of the model. In our study, the DGA < 37 weeks is a risk factor for SPPH, which is consistent with Kim et al. (9) and contrary to Chen et al. (12). It may be associated with more severe conditions in patients who delivered before 37 weeks. Our nomogram includes preoperative HGB < 90 g/L, which is not a predictor in the existing models (9–12). We think that may be related to the amount of RBC transfusion is one of the outcomes of SPPH and the influence of HGB concentration on coagulation function (19, 20). The PB, AP, PP, number of prior CDs, and PAS in the nomogram were similar to the existing models (9–12) and other research (21, 22). More information about the existing models for predicting the risk of SPPH for women with PP is shown in Appendix Table 5.

By comparing the predictive performance of the preoperative nomogram and intraoperative nomogram in the development cohort and validation cohort, we found that the intraoperative nomogram had higher net benefits, calibration, and discrimination than the preoperative nomogram. In addition, the results of the predictor replacement test showed that the intraoperative nomogram possessed acceptable performance when used before operation. Additionally, we can see that the predictive performance improved slightly when VE was replaced by BFS, it may be related to the fact that ultrasound is more sensitive to blood flow than clinician. However, the performance declined slightly when the severity of PAS detected intraoperatively was replaced by ultrasound. It is concluded that there are some deficiencies in judging the severity of PAS by ultrasound, and the more accurate for classifying the severity of PAS preoperatively, the more authentic are the prediction results. It is well known that PAS is a major risk factor for SPPH and patients with PP are at high risk for PAS, so the diagnostic accuracy of the severity of PAS preoperatively should be improved. The methods for predicting the severity of PAS based on ultrasound information are available (23), but their indicators are complex and different sonographers may have subjective differences in judgment of the same indicator, and they have not been widely used. MRI is more accurate than ultrasound in diagnosing the depth and topography of PAS (24). However, MRI is more expensive and has complex imaging principles (25), which limit its general applicability. Nowadays, the researchers try to find biochemical markers of PAS, which may be potential markers for the diagnosis of the severity of PAS. Schwickert A et al. (26) found that the level of VEGF in maternal serum could predict the severity of PAS. Shainker SA et al. (27) found that the soluble VEGF receptor 2, soluble Tie2, median plasminogen activator inhibitor one concentrations, and median antithrombin III concentrations in maternal plasma were associated with the severity of PAS. Yang T et al. (28) found that the hsa-miR-490-3p and hsa-miR-133a-3p were positively correlated not only with the severity of PAS but also with the volume of blood loss during operation. To improve the performance of the prediction model for SPPH, the more convenient and accurate methods for diagnosing the severity of PAS remain to be explored.

After identifying the high-risk group of SPPH, we need to know what preparations should be made preoperatively. We found that the rates of artery balloon presetting, use of tourniquet or serrata forceps, suture for hemostasis, ligation of uterine artery, tamponade balloon or gauze in uterine, hysterectomy, intraoperative cell salvage, and transfusion of other blood products in SPPH group were all higher than non-SPPH group. It indicates that patients with SPPH often need to use a combination of treatment methods, so obstetricians should fully master various hemostatic measures and the transfusion composition ratios and application time preoperatively (4). Referring to the guidelines of cell salvage proposed by the Australia, the United States, and England (29), we recommend cell salvage for patients at high risk of SPPH, but it should be performed by an experienced multidisciplinary team (30). The rates of neonate premature birth, low birth weight, asphyxia, and transfer to neonatology in SPPH group were all higher than the non-SPPH group, so neonatal rescue should be also done well before operation. We hope that the intraoperative nomogram can help clinicians identify the high-risk group and low-risk group of SPPH and make preparation according to the condition. However, a small number of patients without risk factors may experience SPPH (8). Obstetricians should be vigilant when treating patients without risk factors.

There are some strengths in our study. First, it is a multicenter study with a large sample size. Second, we included not only preoperative risk factors but also intraoperative risk factors, which made the predictors more reliable. Third, we tested the intraoperative nomogram and demonstrated its potential value when used preoperatively. There are some limitations in our study. First, it was a retrospective study, all information were collected from the existing data. Second, due to some irresistible reasons, we cannot collect all patients in the sub-research centers and only select the most cases randomly. Third, we included a hospital in the validation cohort that did not appear in the development cohort, but its sample size was small, future studies need to conduct external validation of the developed nomogram.

We developed the preoperative nomogram and intraoperative nomogram to predict the risk of SPPH in women with PP undergoing cesarean delivery. By comparing the discrimination, calibration, and net benefit of the two nomograms in the development cohort and validation cohort, we think that the intraoperative nomogram performed better. Moreover, application of the intraoperative nomogram before operation can still achieve good prediction effect, which can be improved if the severity of PAS can be accurately distinguished preoperatively. We expect to conduct further prospective external validation studies on the intraoperative nomogram to evaluate its application value.

## Data Availability Statement

The raw data supporting the conclusions of this article will be made available by the authors, without undue reservation.

## Ethics Statement

This study was approved by the Ethics Committee of Tongji Hospital, Tongji Medical College, Huazhong University of Science and Technology (TJ-IRB20210738) and granted a waiver of informed consent from participants.

## Author Contributions

XD and HL designed the study. XD, LZ, YB, JX, and HD acquired the data. SW, YL, DD, and SC acquired and analyzed the data. XD performed the statistical analyses, wrote, and submitted the manuscript. XD, HL, LF, and WZ revised the manuscript. All authors contributed to the article and approved the submitted version.

## Conflict of Interest

The authors declare that the research was conducted in the absence of any commercial or financial relationships that could be construed as a potential conflict of interest.

## Publisher's Note

All claims expressed in this article are solely those of the authors and do not necessarily represent those of their affiliated organizations, or those of the publisher, the editors and the reviewers. Any product that may be evaluated in this article, or claim that may be made by its manufacturer, is not guaranteed or endorsed by the publisher.

## References

[1] Jain V Bos H Bujold E. Guideline No. 402: diagnosis and management of placenta previa. J Obstet Gynaecol Can. (2020) 42:906–17. 10.1016/j.jogc.2019.07.019 32591150

[2] Wasim T Bushra N Riaz S Iqbal HI. Fetomaternal outcome in patients with placenta previa. Pak J Med Sci. (2020) 36:952–7. 10.12669/pjms.36.5.1647 32704270 PMC7372655

[3] Gonzalez-Brown V Schneider P. Prevention of postpartum hemorrhage. Semin Fetal Neonatal Med. (2020) 25:101129. 10.1016/j.siny.2020.101129 32782215

[4] Deleu F Deneux-Tharaux C Chiesa-Dubruille C Seco A Bonnet MP. EPIMOMS study group. A population-based analysis of French transfusion practices for women experiencing severe postpartum hemorrhage. Int J Obstet Anesth. (2020) 42:11–9. 10.1016/j.ijoa.2019.07.006 31402309

[5] Centers for Disease Control and Prevention. Pregnancy Mortality Surveillance System. Available online at: http://www.cdc.gov/reproductivehealth/maternalinfanthealth/pmss.html (accessed July 21, 2019).

[6] Shi HF Chen L Wang XX Jiang H Dong S Zhuang Y. Incidence and trend of severe postpartum hemorrhage between 2016 and 2019 in China. Zhonghua Fu Chan Ke Za Zhi. (2021) 56:451–7. 10.3760/cma.j.cn112141-20210209-00070 34304436

[7] Fan D Xia Q Liu L Wu S Tian G Wang W. The incidence of postpartum hemorrhage in pregnant women with placenta previa: a systematic review and meta-analysis. PLoS One. (2017) 12:e0170194. 10.1371/journal.pone.0170194 28107460 PMC5249070

[8] Kawakita T Mokhtari N Huang JC Landy HJ. Evaluation of risk-assessment tools for severe postpartum hemorrhage in women undergoing cesarean delivery. Obstet Gynecol. (2019) 134:1308–16. 10.1097/AOG.0000000000003574 31764744

[9] Kim JW Lee YK Chin JH Kim SO Lee MY Won HS. Development of a scoring system to predict massive postpartum transfusion in placenta previa totalis. J Anesth. (2017) 31:593–600. 10.1007/s00540-017-2365-8 28466102

[10] Lee JY Ahn EH Kang S Moon MJ Jung SH Chang SW. Scoring model to predict massive post-partum bleeding in pregnancies with placenta previa: a retrospective cohort study. J Obstet Gynaecol Res. (2018) 44:54–60. 10.1111/jog.13480 29067758

[11] Kang J Kim HS Lee EB Moon MJ Jung SH Chang SW. Prediction model for massive transfusion in placenta previa during cesarean section. Yonsei Med J. (2020) 61:154–60. 10.3349/ymj.2020.61.2.154 31997624 PMC6992462

[12] Chen C Liu X Chen D Huang S Yan X Liu H. A risk model to predict severe postpartum hemorrhage in patients with placenta previa: a single-center retrospective study. Ann Palliat Med. (2019) 8:611–21. 10.21037/apm.2019.09.04 31594367

[13] Kleinrouweler CE Cheong-See FM Collins GS Kwee A Thangaratinam S Khan KS. Prognostic models in obstetrics: available, but far from applicable. Am J Obstet Gynecol. (2016) 214:79–90.e36. 10.1016/j.ajog.2015.06.013 26070707

[14] Neary C Naheed S McLernon DJ Black M. Predicting risk of postpartum haemorrhage: a systematic review. BJOG. (2021) 128:46–53. 10.1111/1471-0528.16379 32575159

[15] Jauniaux E Ayres-de-Campos D, FIGO Placenta Accreta Diagnosis and Management Expert Consensus Panel. FIGO consensus guidelines on placenta accreta spectrum disorders: introduction. Int J Gynaecol Obstet. (2018) 140:261–4. 10.1002/ijgo.12406 29405322

[16] Pacheco LD Saade GR Costantine MM Clark SL Hankins GD. An update on the use of massive transfusion protocols in obstetrics. Am J Obstet Gynecol. (2016) 214:340–4. 10.1016/j.ajog.2015.08.068 26348379

[17] Seligman K Ramachandran B Hegde P Riley ET El-Sayed YY Nelson LM. Obstetric interventions and maternal morbidity among women who experience severe postpartum hemorrhage during cesarean delivery. Int J Obstet Anesth. (2017) 31:27–36. 10.1016/j.ijoa.2017.03.009 28676403 PMC5578415

[18] Park SY. Nomogram: an analogue tool to deliver digital knowledge. J Thorac Cardiovasc Surg. (2018) 155:1793. 10.1016/j.jtcvs.2017.12.107 29370910

[19] Nair M Chhabra S Choudhury SS Deka D Deka G Kakoty SD. Relationship between anaemia, coagulation parameters during pregnancy and postpartum haemorrhage at childbirth: a prospective cohort study. BMJ Open. (2021) 11:e050815. 10.1136/bmjopen-2021-050815 34607867 PMC8491293

[20] Omotayo MO Abioye AI Kuyebi M Eke AC. Prenatal anemia and postpartum hemorrhage risk: a systematic review and meta-analysis. J Obstet Gynaecol Res. (2021) 47:2565–76. 10.1111/jog.14834 34002432 PMC9258034

[21] Liu CN Yu FB Xu YZ Li JS Guan ZH Sun MN. Prevalence and risk factors of severe postpartum hemorrhage: a retrospective cohort study. BMC Pregnancy Childbirth. (2021) 21:332. 10.1186/s12884-021-03818-1 33902475 PMC8077797

[22] Du L Feng L Bi S Zhang L Tang J Zhong L. Probability of severe postpartum hemorrhage in repeat cesarean deliveries: a multicenter retrospective study in China. Sci Rep. (2021) 11:8434. 10.1038/s41598-021-87830-7 33875708 PMC8055978

[23] Chong Y Zhang A Wang Y Chen Y Zhao Y. An ultrasonic scoring system to predict the prognosis of placenta accreta: a prospective cohort study. Medicine. (2018) 97:e12111. 10.1097/MD.0000000000012111 30170439 PMC6392640

[24] Jha P Poder L Bourgioti C Bharwani N Lewis S Kamath A. Society of Abdominal Radiology (SAR) and European Society of Urogenital Radiology (ESUR) joint consensus statement for MR imaging of placenta accreta spectrum disorders. Eur Radiol. (2020) 30:2604–15. 10.1007/s00330-019-06617-7 32040730

[25] Kapoor H Hanaoka M Dawkins A Khurana A. Review of MRI imaging for placenta accreta spectrum: Pathophysiologic insights, imaging signs, and recent developments. Placenta. (2021) 104:31–9. 10.1016/j.placenta.2020.11.004 33238233

[26] Schwickert A Chantraine F Ehrlich L Henrich W Muallem MZ Nonnenmacher A. Maternal serum VEGF predicts abnormally invasive placenta better than NT-proBNP: a multicenter case-control study. Reprod Sci. (2021) 28:361–70. 10.1007/s43032-020-00319-y 33025531 PMC7808970

[27] Shainker SA Silver RM Modest AM Hacker MR Hecht JL Salahuddin S. Placenta accreta spectrum: biomarker discovery using plasma proteomics. Am J Obstet Gynecol. (2020) 223:433.e1–e14. 10.1016/j.ajog.2020.03.019 32199927

[28] Yang T Li N Hou R Qiao C Liu C. Development and validation of a four-microRNA signature for placenta accreta spectrum: an integrated competing endogenous RNA network analysis. Ann Transl Med. (2020) 8:919. 10.21037/atm-20-1150 32953719 PMC7475428

[29] Dahlke JD Mendez-Figueroa H Maggio L Hauspurg AK Sperling JD Chauhan SP. Prevention and management of postpartum hemorrhage: a comparison of 4 national guidelines. Am J Obstet Gynecol. (2015) 213:76.e1–76.e10 10.1016/j.ajog.2015.02.023 25731692

[30] Klein AA Bailey CR Charlton AJ Evans E Guckian-Fisher M McCrossan R. Association of anaesthetists guidelines: cell salvage for peri-operative blood conservation. Anaesthesia. (2018) 73:1141–50. 10.1111/anae.14331 29989144

