# Supplementary material for: Developing and Validating Nomogram to Predict Severe Postpartum Hemorrhage in Women With Placenta Previa Undergoing Cesarean Delivery: A Multicenter Retrospective Case-Control Study
Source: Front Med (Lausanne). 2022 Feb 11;8:789529. doi: 10.3389/fmed.2021.789529 (PMC8873861; doi:10.3389/fmed.2021.789529)
Supplement: Supplementary file 1 [file Table_2.DOCX]

**Appendix table 1 Details of the research centers**

| Recruitment site | Principal investigator | The number of patients recruited | |
| --- | --- | --- | --- |
|  |  | Development cohort（n=937） | Validation Cohort(n=313) |
| Tongji Hospital | Xiaohe Dang | 542 | 144 |
| The Central Hospital of Wuhan | Li Zhang | 112 | 52 |
| Renmin Hospital of Wuhan University | Yindi Bao | 159 | 38 |
| Xianning Central Hospital | Jie Xu | 124 | 57 |
| Maternal and Child Health Hospital of Hubei Province | Hui Du | non | 22 |

Due to the small sample of the validation cohort, 22 patients were recruited from Maternal and Child Health Hospital of Hubei Province.

**Appendix table 2 Preoperative and intraoperative judgment of the severity of placenta accreta spectrum (PAS)**

|  | Development cohort（n=865） | | Validation Cohort(n=307) | |
| --- | --- | --- | --- | --- |
| Type | Preoperative ultrasound | Intraoperative | Preoperative ultrasound | Intraoperative |
| Normal-n (%) | 660 (76.30) | 426 (49.25) | 232 (75.57) | 132 (42.30) |
| Crete-n (%) | 11 (1.27) | 142 (16.42) | 5 (1.63) | 77 (25.08) |
| Increta-n (%) | 186 (21.50) | 276 (31.91) | 69 (22.48) | 90 (29.31) |
| Percreta-n (%) | 8 (0.92) | 21 (2.43) | 1 (0.33) | 8 (2.61) |

**Appendix table 3 Intraoperative hemostasis measures of the development cohort and validation cohort**

|  | Development cohort（n=865） | | | Validation cohort（n=307） | | |
| --- | --- | --- | --- | --- | --- | --- |
| Measures | Non SPPH group  (n=661) | SPPH group  (n=204) | *P-*value | Non SPPH group  (n=227) | SPPH group  (n=80) | *P-*value |
| Arterial balloon presetting-n (%) | 9 (1.36) | 10 (4.90) | 0.006 | 0 (0.00) | 4 (5.00) | 0.005 |
| Tourniquet or Serrata forceps-n (%) | 15 (2.27) | 32 (15.69) | ＜0.001 | 10 (4.41) | 24 (30.00) | ＜0.001 |
| Uterine artery ligation-n (%) | 60 (9.08) | 69 (7.98) | ＜0.001 | 17 (7.49) | 45 (56.25) | ＜0.001 |
| Suture hemostasis-n (%) | 311 (47.05) | 177 (86.76) | ＜0.001 | 116 (51.10) | 71 (88.75) | ＜0.001 |
| Balloon or gauze filling-n (%) | 13 (1.97) | 27 (13.24) | ＜0.001 | 3 (1.32) | 10 (12.50) | ＜0.001 |
| Hysterectomy-n (%) | 0 (0.00) | 12 (5.88) | ＜0.001 | 0 (0.00) | 4 (5.00) | 0.005 |
| Cell salvage-n (%) | 6 (0.91) | 42 (20.59) | ＜0.001 | 6 (2.64) | 27 (33.75) | ＜0.001 |
| Other blood products (platelets, fibrin, plasma et al.) -n (%) | 30 (4.54) | 124 (60.78) | ＜0.001 | 6 (2.64) | 27 (33.75) | ＜0.001 |

**Appendix table 4 Maternal and neonate prognosis of the** **development cohort and validation cohort**

|  | **Development cohort（n=865）** | | | **Validation cohort（n=307）** | | |
| --- | --- | --- | --- | --- | --- | --- |
| **Maternal** | **Non SPPH group**  **(n=661)** | **SPPH group**  **(n=204)** | ***P*-value** | **Non SPPH group**  **(n=227)** | **SPPH group**  **(n=80)** | ***P*-value** |
| Postoperative bleeding-n (%) | 1 (0.15) | 11 (5.39) | ＜0.001 | 1 (0.44) | 2 (2.50) | 0.167 |
| ICU-n (%) | 0 (0.00) | 26 (12.75) | ＜0.001 | 0 (0.00) | 2 (2.50) | 0.067 |
| Postoperative  hospital stay（d） | 5 (4,6) | 6 (4,9) | ＜0.001 | 5 (4, 6) | 6 (4, 10) | ＜0.001 |
| **Neonate** |  |  |  |  |  |  |
| Premature (＜37w)-n (%) | 331 (50.07) | 161 (78.92) | ＜0.001 | 102 (44.93) | 64 (80.00) | ＜0.001 |
| Weight＜2500g-n (%) | 149 (22.54) | 69 (33.82) | 0.001 | 43 (18.94) | 30 (37.50) | 0.001 |
| 1-min Apgar score ≤7’ -n (%) | 114 (17.25) | 66 (32.35) | ＜0.001 | 23 (10.13) | 24 (30.00) | ＜0.001 |
| 5-min Apgar score ≤7’ -n (%) | 24 (3.63) | 17 (8.33) | 0.006 | 6 (2.64) | 9 (11.25) | 0.006 |
| Transfer to neonatology-n (%) | 155 (23.45) | 86 (42.16) | ＜0.001 | 58 (25.55) | 38 (47.50) | ＜0.001 |

**Appendix table 5 The existing models for predicting the risk of severe postpartum hemorrhage (SPPH) for women with placenta previa**

| Author (year of publication) | SPPH definition | Model |
| --- | --- | --- |
| Kim JW et al. (2017) | Red blood cell transfusion≥8U | Total points= preoperative suspicion of placental adhesion (2 points) +times of previous caesarean section (2:2 points; 1:1 points; 0:0 points) +gestational age < 37 weeks (1 point) +anterior placenta (1 point) +sponge-like appearance of cervix (1 point) |
| Lee JY et al. (2018) | Blood loss ≥2000mL or red blood cell transfusion ≥4U or uterine artery embolization or hysterectomy | Total points=maternal age ≥ 35 (1 points) +antepartum hemorrhage (2 points) + fetal non-cephalic presentation (2 points) + complete placenta previa (1 points) + anterior placenta (1 points) + uteroplacental hypervascularity (2 points) + multiple lacunae ≥ 4 (1 points) |
| Kang Jieun et al. (2020) | Red blood cell transfusion ≥5U | P=Log(Y/1−Y)  Y=-25.615+0.099×maternal age+19.253×completely or partially placenta previa+0.210×marginal placenta previa +17.035×low-lying placenta +0.118×lacunae1+0.441×lacunae2+1.256×lacunae3+2.003×hypoechoic layer+1.404×anterior placenta  (lacunae grade as follows: 0 for no lacuna was detected; 1 for 1–3 generally small lacunae were present;2 for 4–6 larger or more irregular lacunae were present; and 3 for many lacunae detected throughout the placenta, some appearing large and irregular in shape. And the equations are presented in the form of nomogram.） |
| Chen C et al. (2019) | Blood loss ≥1500ml | P=Log(Y/1−Y)  Y=−6.942+0.075×maternal age+1.531×times of delivery+0.223×times of miscarriage−3.557×history of vaginal delivery (1 for yes, 0 for no) + 1.753× gestational age (0 for <37 weeks, 1 for ≥37 weeks) +1.574× relationship between placenta and uterine muscle wall according to ultrasound data (0 for normal, 1 for placenta adhesion, 2 for placenta implantation, 3 for placenta penetration) |
